# Lipid droplets promote efficient mitophagy

**DOI:** 10.1080/15548627.2022.2089956

**Published:** 2022-08-08

**Authors:** Maeve Long, Thomas G. McWilliams

**Affiliations:** a Translational Stem Cell Biology & Metabolism Program, Research Programs Unit, Faculty of Medicine, Biomedicum Helsinki, University of Helsinki, Helsinki, Finland; b Department of Anatomy, Faculty of Medicine, Biomedicum Helsinki, University of Helsinki, Helsinki, Finland

**Keywords:** DGAT1, iron, lipid droplet, metabolism, mitophagy

## Abstract

Mitophagy neutralizes defective mitochondria *via* lysosomal elimination. Increased levels of mitophagy hallmark metabolic transitions and are induced by iron depletion, yet its metabolic basis has not been studied in-depth. How mitophagy integrates with different homeostatic mechanisms to support metabolic integrity is incompletely understood. We examined metabolic adaptations in cells treated with deferiprone (DFP), a therapeutic iron chelator known to induce PINK1-PRKN-independent mitophagy. We found that iron depletion profoundly rewired the cellular metabolome, remodeling lipid metabolism within minutes of treatment. DGAT1-dependent lipid droplet biosynthesis occurs upstream of mitochondrial turnover, with many LDs bordering mitochondria upon iron chelation. Surprisingly, DGAT1 inhibition restricts mitophagy *in vitro* by lysosomal dysfunction. Genetic depletion of mdy/DGAT1 *in vivo* impairs neuronal mitophagy and locomotor function in *Drosophila*, demonstrating the physiological relevance of our findings.

Starvation provides a classic stimulus to study nonselective macroautophagy/autophagy, yet pioneering studies of GFP-LC3 reporter mice reveal instances of steady state physiological autophagy in several mammalian tissues. Similarly, the advent of mitophagy reporter mice revealed variegated landscapes of steady-state mitochondrial destruction in mammals. Most of our mitophagy knowledge derives from meticulous work on PINK1-PRKN signaling, a depolarization-dependent stress pathway that promotes mitochondrial ubiquitination. Yet the evolutionarily conserved process of physiological mitophagy proceeds independently of the PINK1-PRKN pathway. Thus, there is a clear need to identify factors that promote or prevent physiological mitophagy in a variety of contexts.

Hypoxia and iron depletion are widely used to study PINK1-PRKN-independent mitophagy, and both stimuli share similarities by promoting cellular glycolytic dependence and HIF1A-stabilization. Studies of iron depletion-induced mitophagy have traditionally focused on treatment time points corresponding to the maximal amount of mitochondrial turnover (typically “overnight” exposures exceeding 18 h). These studies have proven invaluable for the discovery of numerous “*eat me*” signals and regulatory mechanisms of selective autophagy. Iron is a tightly regulated cofactor, and its extended depletion induces an array of additional phenotypes, including loss of mitochondrial respiration and lipid droplet (LD) accumulation. Carbon substitution experiments (whereby glucose is switched to galactose) inhibit mitophagy, highlighting the importance of metabolic state for DFP-induced mitophagy. Given the intriguing relationship between iron, mitochondria and metabolism, steady-state or basal mitophagy may also provide an important means for liberation or redistribution of iron *in vivo*.

Our study profiles the metabolic events preceding PINK1-PRKN-independent mitophagy, using the highly defined approach afforded by iron depletion [1]. We performed temporal metabolomics on human ARPE19 cells treated with DFP at a range of acute and extended time points, ranging from 15 min to 48 h. We found that iron depletion rapidly reshapes the cellular metabolome. Specifically, iron depletion induces alterations in lipid composition, with carnitine homeostasis and sterol metabolism affected by short DFP exposures. Additional analyses revealed lipid metabolic pathways are highly affected, with “decreased oxidation of branched fatty acids” and “increased *de novo* triacylglycerol biosynthesis” among the top affected pathways. Altered lipid metabolism was also verified by differential gene expression signatures for carnitine synthesis, fatty acid metabolism, sterol biosynthesis and glycogen synthesis.

These changes are accompanied by the accumulation of neutral LDs, which are located proximal to the mitochondrial network at time points preceding mitophagy. Prior work identified LD formation following sustained iron depletion, yet the biogenesis mechanism and its relationship to selective autophagy had not been studied. Using pharmacological inhibitors and RNAi-mediated depletion, we determined that DGAT1 (diacylglycerol O-acyltransferase 1) promotes LD biogenesis upon iron chelation in various cell types. A well-defined link exists between macroautophagy and LD biogenesis: macroautophagy liberates fatty acids, and some of these are re-esterified and packaged into nascent LDs, thereby protecting mitochondria from lipotoxicity. In this context, impaired autophagic flux inhibits LD biogenesis. We explored the requirement for autophagy signaling in iron-depletion LD biogenesis using *ULK1* knockout cells. DFP promotes LD accumulation in the absence of autophagy signaling, and is not noticeably different upon re-complementation with wild-type FLAG-ULK1.

LDs are much more than fat storage organelles and reportedly contribute to autophagosome biogenesis in certain contexts. We next examined the relationship between DGAT1-dependent LD biosynthesis and mitophagy. Pharmacogenetic inhibition of DGAT1 in numerous *mito*-QC reporter cell lines reveals decreased mitolysosome abundance. This defect is not a consequence of impaired damage sensing, as iron depletion readily induces mitochondrial BNIP3L/NIX accumulation in the absence of LDs. We also observed robust formation of ULK1 and WIPI2 foci irrespective of LD inhibition, demonstrating no impairment in autophagy initiation signals. Mechanistically, impaired LD biogenesis does not affect damage sensing, priming or autophagic induction upon iron depletion. Impaired LD biogenesis can promote the cytotoxic accumulation of non-esterified or free fatty acids, which affect lysosomal activity and pH. In agreement with this, loss of DGAT1 activity leads to altered positioning of cathepsin-positive endolysosomes upon iron chelation. The position of lysosomes underpins their function and thus we predict their altered homeostasis accounts for the reduced mitophagy levels observed in *vitro*. The combined loss of DGAT1-dependent LD biosynthesis and iron depletion also exacerbate mitochondrial damage, lipotoxicity and reduced cell survival. Because other mitophagy stimuli do not promote LD accumulation, it is likely this mechanism is context dependent.

Previous work has shown basal mitophagy is conserved from mammals to flies. DGAT1 has a single ortholog in *Drosophila* termed mdy (midway), which controls LD biogenesis and homeostasis *in vivo*. We verified the physiological relevance of DGAT1 in mitophagy by phenotyping reporter *Drosophila* lines deficient for mdy. Targeted *mdy* depletion using previously characterized inducible RNAi transgenes and drivers revealed an important role for *mdy* in development, consistent with its critical metabolic function. Previous studies of *mito*-QC mitophagy reporter flies identified high levels of mitochondrial turnover in neurons; thus, we analyzed the effects of *mdy* depletion on neuronal mitophagy. We employed a distinct reporter strategy for our *in vivo* studies, placing the mCherry-GFP tag in the mitochondrial matrix (*matrix*-QC, generated and characterized by the Alex Whitworth lab, Cambridge). Pan-neuronal depletion of *mdy* in *matrix*-QC reporter animals significantly reduces neuronal mitophagy in both *mdy* knockdown conditions. Mitolysosomes in *mdy*-deficient animals are less abundant and are altered in profile compared to control counterparts. In some animals, the effects on mitolysosomes are accompanied by developmental defects in wing posture similar to those observed in *pink1* and *park* (parkin) mutant flies. Strikingly, neuron-specific *mdy* depletion induces locomotor dysfunction, compromising climbing behavior in flies. These *in vivo* experiments establish that the balance of LDs is sufficient to modify physiological mitophagy, further highlighting their emerging importance for tissue integrity and development.

In summary, we find that iron depletion affects metabolism more rapidly than previously appreciated, and redirects glycolytic flux toward adaptive lipid biosynthesis to preserve organelle and cellular integrity. We identify a novel role for DGAT-mediated LD crosstalk in mitochondrial turnover, which appears important in a highly defined context of basal mitophagy *in vivo*. Further dissection of this and additional crosstalk mechanisms will prove useful for targeting mitophagy in disease.
